# Differentiating Systemic Lupus Erythematosus Flare From Preeclampsia in Pregnancy Using the Soluble Fms-Like Tyrosine Kinase-1/Placental Growth Factor Ratio

**DOI:** 10.7759/cureus.83619

**Published:** 2025-05-06

**Authors:** Akane Yamamoto, Manabu Ogoyama, Akihide Ohkuchi, Hironori Takahashi, Hiroyuki Fujiwara

**Affiliations:** 1 Department of Obstetrics and Gynecology, Jichi Medical University, Shimotsuke, JPN

**Keywords:** preeclampsia, pregnancy, proteinuria, sflt-1/plgf ratio, systemic lupus erythematosus

## Abstract

Systemic lupus erythematosus (SLE) may be exacerbated at any stage of pregnancy, complicating maternal and fetal outcomes. Additionally, pregnancies with SLE have a higher risk of preeclampsia (PE), requiring careful differentiation between SLE flare and PE when symptoms such as proteinuria emerge. We herein describe a 36-year-old pregnant woman with SLE who developed severe proteinuria (13 g/day) at 30 weeks of gestation without hypertension or thrombocytopenia. No abnormal urinary segments were observed. The differential diagnosis between SLE flare and the preliminary sign of PE was challenging. The soluble fms-like tyrosine kinase-1 (sFlt-1)/placental growth factor (PlGF) ratio was utilized for diagnostic clarification. A normal sFlt-1/PlGF ratio at 29+4 weeks of gestation suggested SLE flare rather than the preliminary sign of PE. Intensified immunosuppressive therapy with increased prednisolone (30 mg/day) attenuated proteinuria, allowing for late-term pregnancy. At 37+4 weeks of gestation, the patient developed late-onset PE with a hypertensive crisis, necessitating emergency cesarean delivery. The infant was delivered safely without complications. Postpartum recovery was uneventful, with stable maternal renal function. This case underscores the importance of angiogenic markers in distinguishing SLE flare from PE. An elevated sFlt-1/PlGF ratio is typically associated with PE, but not SLE flare, aiding in a differential diagnosis and guiding treatment strategies.

## Introduction

In pregnancies complicated by systemic lupus erythematosus (SLE), disease activity can worsen at any stage, with reported flare rates ranging from 15% to 85% [1]. Therefore, pregnancy in SLE patients should be approached with caution, and it is recommended that the following criteria be met: inactive lupus nephritis, proteinuria < 0.5 g/day, preserved renal function (glomerular filtration rate > 60 mL/min/1.73 m²), and disease control maintained with medications deemed safe for use during pregnancy [2-4]. Recommended treatment options include hydroxychloroquine, an antimalarial agent, and immunosuppressive drugs, such as oral glucocorticoids, azathioprine, cyclophosphamide, and tacrolimus, all of which have been reported to effectively suppress SLE activity during pregnancy [5,6]. More recently, belimumab, a human anti-B lymphocyte stimulator monoclonal antibody, has shown promise in pregnant women with active SLE, yielding favorable maternal and fetal outcomes [7,8]. Therefore, even when SLE flare occurs during pregnancy, continuation of pregnancy may be possible with appropriate management. Conversely, pregnancies complicated by SLE carry an increased risk of preeclampsia (PE), which is characterized by hypertension with proteinuria or other organ dysfunction after 20 weeks of gestation, with a reported incidence of approximately 20%-30% [6,9]. The only definitive treatment for PE is termination of the pregnancy. In cases of early-onset PE, termination may be unavoidable; however, considering the risks of neonatal complications due to fetal immaturity, premature delivery needs to be carefully assessed. Therefore, in pregnancies complicated by SLE, it is crucial to establish whether findings such as the exacerbation of proteinuria and thrombocytopenia are due to PE, SLE flare, or lupus nephritis. We herein present a case of pregnancy complicated by SLE in which severe proteinuria developed with minimal accompanying symptoms, necessitating differentiation between the preliminary sign of PE and SLE flare. In this case, an evaluation of the soluble fms-like tyrosine kinase-1 (sFlt-1)/placental growth factor (PlGF) ratio was highly beneficial.

## Case presentation

**Figure 1:**
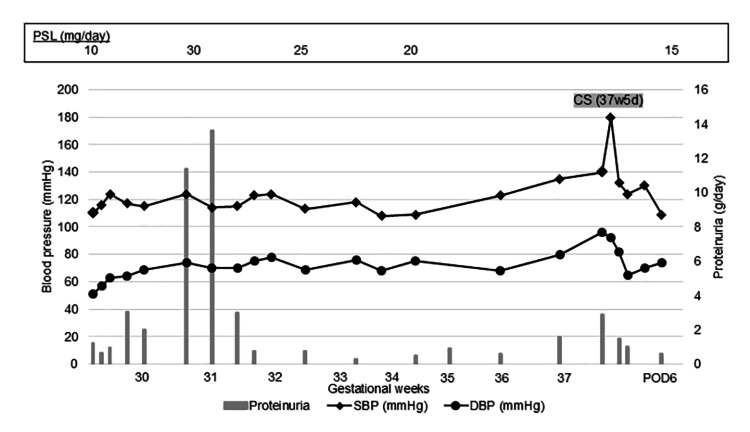
Changes in blood pressure and urine protein content Urine protein content included the value of stored urine protein or the urine protein-to-creatinine ratio. CS: cesarean section, DBP: diastolic blood pressure, POD: postoperative day, PSL: prednisolone, SBP: systolic blood pressure.

The patient was diagnosed with SLE at the age of 26 based on the presence of fever, pancytopenia, progressive glomerulonephritis, hypocomplementemia, and positive antinuclear antibodies (ANAs). She was managed with prednisolone (10 mg/day) and tacrolimus (3 mg/day). Her urine protein remained below 0.5 g/day, and renal function was preserved (estimated glomerular filtration rate > 100 mL/min/1.73 m²). Based on this stable disease status, her attending physician approved pregnancy, and she conceived naturally at the age of 36. At 16 weeks of gestation, she was referred to our institution as a high-risk pregnancy due to underlying SLE. Initial evaluation showed normal blood pressure (113/65 mmHg) and renal function (serum creatinine: 0.52 mg/dL). However, proteinuria was present (++), with a urine protein-to-creatinine ratio (UPCR) of 1.2 g/g·Cr. Autoantibody testing revealed positive ANA (speckled type, ×320), anti-double-stranded (ds) DNA antibody (3.2 IU/mL), anti-SS-A antibody (×16), anti-Sm antibody (×8), and anti-CCP antibody (0.5 U/mL), with negative results for anti-SS-B antibody. She was a primipara with no history of PE. Because she was referred at 16 weeks of gestation, low-dose aspirin for preterm PE prevention was not initiated. Throughout the pregnancy, fetal growth remained normal, and no fetal bradyarrhythmia (such as atrioventricular block) was observed. Maternal blood pressure remained stable, while proteinuria persisted at a UPCR of approximately 1.0 g/g·Cr. Figure 1 displays the progression of blood pressure and proteinuria during pregnancy, and Table 1 summarizes changes in serum biochemical parameters.

**Table 1:** Changes in serum biochemical data ALT: alanine aminotransferase, AST: aspartate aminotransferase, CS: cesarean section, dsDNA: double-stranded deoxyribonucleic acid, eGFR: estimated glomerular filtration rate, PlGF: placental growth factor, POD: postoperative day, PSL: prednisolone, sFlt-1: soluble fms-like tyrosine kinase-1, w: weeks, d: days.

	27w5d	30w4d	31w3d	32w4d	33w3d	34w3d	35w6d	36w6d	37w4d	37w5d	POD1	POD4
Event			▼ PSL 30 mg/day		▼ PSL 25 mg/day		▼ PSL 20 mg/day			▼ CS		
C3 (mg/dL)	109	127	113		104	92			89			
C4 (mg/dL)	20	29	25		20	17			16			
CH50 (U/mL)	52.1	63.1	55.2		53.3	49.1			47.6			
Anti-dsDNA IgG (IU/mL)	3.2	25.8	5.7		4.4	8			8.3			
Platelets (×10^4^/μL)		18.0		24.8	18.8	19.1	22.2	22.1	22.3	22.5	21.4	22.6
Antithrombin III (%)			75.6	95.9	102.8	95.7	93.9	83.7	93.5	98.6	86.1	
Creatinine		0.52	0.50	0.50	0.54	0.57	0.64	0.68	0.64	0.66	0.50	0.56
eGFR (mL/min/1.73 m^2^)		105		109			84	78	84	81	109	
Uric acid (mg/dL)		4.7	5.2	4.3	4.3	4.6	5.7	6.3	6.4	6.5	4.2	
Total bilirubin (mg/dL)		0.67	0.42	0.68	0.69	0.88	0.72	0.87	0.68	0.89		0.54
AST (U/L)		48	211	318	483	865	562	396	292	295	210	103
ALT (U/L)		42	281	469	708	1258	677	452	467	455	379	229
sFlt-1/PlGF ratio		1.7	4.4									

During a routine prenatal checkup at 29+4 weeks of gestation, the patient's blood pressure remained within the normal limits; however, proteinuria had worsened (UPCR: 3.0 g/g·Cr), along with significant weight gain (+1.9 kg/two weeks). She was admitted for close monitoring. At 30+0 weeks, a vesicular rash appeared on the right occipital scalp and was diagnosed as herpes zoster, rather than an SLE-associated rash. Oral amenamevir (400 mg/day) was initiated. By 30+4 weeks, proteinuria had markedly increased to 11.4 g/day. However, there were no accompanying thrombocytopenia, hypocomplementemia, or abnormal urinary sediment, and no other significant signs of SLE flare or lupus nephritis were observed. Additionally, maternal blood pressure remained stable, and a definitive diagnosis of PE could not be established. At this stage, it was unclear whether worsening proteinuria resulted from SLE flare or the prodrome of PE. The sFlt-1/PlGF ratio, measured on admission (29+4 weeks of gestation), was low at 1.7, suggesting that the condition was more consistent with SLE flare than PE-related nephropathy. Consequently, in a consultation with the attending rheumatologist, the findings were deemed atypical for SLE exacerbation, but since there was no significant increase in blood pressure and her condition did not require immediate termination of pregnancy, we decided to first try increasing the dose of prednisolone. The oral prednisolone dose was increased to 30 mg/day. If the patient’s condition fails to improve, further escalation with agents such as belimumab or intensified monitoring for PE would be considered. Following the dose escalation of prednisolone, proteinuria peaked at 13.7 g/day at 31+0 weeks of gestation and subsequently declined to 6.5 g/day at 31+3 weeks and further to 1.0 g/day at 32+0 weeks of gestation. During this period, maternal blood pressure remained stable, and fetal well-being was preserved. A follow-up sFlt-1/PlGF ratio at 31+5 weeks of gestation was low at 4.4. Although the result of the anti-dsDNA antibody level measured at 30+4 weeks of gestation (when prednisolone was increased) was not immediately available, subsequent results showed a peak at 25.8 U/mL, which decreased with treatment. Around the time of herpes zoster, the patient simultaneously exhibited a transient elevation in liver transaminases, with peak levels of aspartate aminotransferase (1,258 U/L) and alanine aminotransferase (865 U/L). Ursodeoxycholic acid was initiated for hepatoprotection. Liver ultrasound showed no significant abnormalities, and serological testing for autoimmune hepatitis (i.e., anti-mitochondrial antibody) and viral infections (Epstein‐Barr virus and others) was negative. In the absence of a clear etiology, drug-induced or pregnancy-related hepatic dysfunction was suspected, and careful monitoring was continued. Since the patient’s condition remained stable, she was transitioned to outpatient management with weekly follow-up after 34 weeks of gestation. Liver enzyme levels gradually normalized, and her rash on the head showed improvement during follow-up.

During a prenatal checkup at 36+6 weeks of gestation, the patient exhibited high-normal blood pressure (130/80 mmHg) and worsening proteinuria (UPCR: 1.6 g/g·Cr), prompting readmission. At 37+4 weeks of gestation, she experienced premature rupture of membranes (PROM) just prior to the planned hospital readmission. The amniotic fluid was clear. On admission, blood pressure had risen to 140/96 mmHg, and UPCR had increased to 2.9 g/g·Cr, leading to a diagnosis of late-onset PE. Empiric antibiotic therapy with flomoxef was initiated for infection prophylaxis. Labor induction was scheduled; however, the following day, the patient developed a hypertensive crisis with blood pressure reaching 180/92 mmHg. Intravenous magnesium sulfate and continuous nicardipine infusion were initiated for blood pressure control and seizure prophylaxis. Concurrently, severe, prolonged decelerations were observed on fetal heart rate monitoring, indicating fetal distress. Due to the worsening maternal hypertension and evidence of fetal compromise, an emergency cesarean section was performed under general anesthesia due to persistent fetal bradycardia. A male infant weighing 2,727 g was delivered with Apgar scores of 8 (1 minute) and 9 (5 minutes), and an umbilical arterial pH of 7.237. Fetal bradycardia was suspected to be due to umbilical cord compression, likely secondary to oligohydramnios following PROM. A histopathological examination of the placenta revealed severe chorioamnionitis; however, the characteristic findings associated with hypertensive disorders of pregnancy, such as placental infarctions, were absent. On postoperative day (POD) 1, intravenous magnesium sulfate and nicardipine were discontinued, and antihypertensive therapy was transitioned to oral nifedipine (20 mg/day). Subsequently, blood pressure remained within normal to borderline-high levels. The amelioration of proteinuria was noted, with a UPCR of 0.6 g/day noted by POD6. Both the mother and the infant recovered uneventfully and were discharged on POD7. Post-discharge, the patient continued regular follow-ups with her rheumatologist, and prednisolone tapering was initiated.

## Discussion

In the present case, proteinuria increased to 13 g/day during pregnancy complicated by SLE. However, the absence of hypertension, thrombocytopenia, and complement depletion made it difficult to distinguish between an SLE flare and early-onset PE. As the sFlt-1/PlGF ratio did not increase, we concluded that the underlying pathology was an SLE flare rather than PE. Therefore, instead of terminating the pregnancy, we intensified the SLE treatment, which reduced proteinuria and allowed the pregnancy to continue successfully to term.

The ratio of sFlt-1, a soluble vascular endothelial growth factor (VEGF) receptor, to PlGF is widely used to predict the onset and severity of PE. The sFlt-1/PlGF ratio is particularly useful for the exclusion diagnosis of PE. Specifically, when the ratio is ≤38, the probability of developing PE within one week is less than 2%, whereas a ratio >38 is associated with a 30%-40% probability of PE onset within four weeks [10,11]. In the present case, the sFlt-1/PlGF ratio at the time of hospitalization (29+4 weeks of gestation) was 1.4, which was below the cutoff value. This indicated an extremely low probability of PE developing within one week, supporting our decision that the exacerbation of proteinuria (13 g/day at 30+4 weeks of gestation) was unlikely to be related to PE. Additionally, recent studies suggested that the sFlt-1/PlGF ratio serves as a predictive marker for severe PE and the need for immediate delivery due to PE-related complications (e.g., placental abruption, hepatic or renal dysfunction). Specifically, cutoff values ≥85 before 36 weeks and ≥110 after 36 weeks are considered useful for predicting imminent delivery due to PE-related complications [12,13]. On the other hand, while proteinuria may also be exacerbated during SLE flare and/or lupus nephritis, sFlt-1/PlGF does not increase in this setting, distinguishing it from the course of PE. For example, Mayer-Pickel et al. measured serum angiogenic factors every four weeks after 12 weeks of gestation in pregnant women with SLE (n = 23) and antiphospholipid syndrome (n = 35) [14]. The findings demonstrated that those who later developed early-onset PE had significantly higher sFlt-1/PlGF ratios at all time points than those who did not. Moreover, among individual angiogenic factors, sFlt-1 and soluble endoglin (sEng) levels were significantly elevated, whereas PlGF levels were significantly lower in women who developed early-onset PE. Similarly, Leaños-Miranda et al. compared pregnant women with SLE who developed PE (n = 42) and those who did not (n = 75) and found similar changes in serum angiogenic factors beyond 12 weeks of gestation [15]. In the present study, a high sFlt-1/PlGF ratio at 12 weeks was associated with the subsequent development of early-onset PE, while elevated levels beyond 24 weeks were associated with late-onset PE. Furthermore, de Jesús et al. conducted a comparative study among three groups of pregnant women: those with inactive SLE (SLEPDAI score < 4, n = 41), those with active lupus nephritis (SLEPDAI score > 4 including renal criteria, n = 15), and those with SLE who developed PE (n = 15) [16]. Their findings revealed that pregnant women with SLE who developed PE had significantly higher sFlt-1 levels, lower PlGF levels, and an elevated sFlt-1/PlGF ratio than the other groups. Moreover, their study indicated that VEGF levels were significantly higher in pregnant women with active lupus nephritis, suggesting its potential as a novel biomarker for differentiation. We previously encountered cases that support these findings. One case involved a pregnant woman with SLE, in which hypertension and proteinuria became apparent after 25 weeks of gestation, raising the suspicion of PE. However, the pregnancy was prolonged until 33 weeks, at which point renal function deteriorated [17]. Serum creatinine and proteinuria both continued to increase postpartum, prompting renal biopsy, which confirmed a diagnosis of diffuse lupus nephritis (class IV-G(A)). A retrospective analysis revealed that the maternal serum sFlt-1/PlGF ratio at 27 and 28 weeks of gestation remained within normal limits, which reinforced that this pregnancy course was not due to PE. Another case involved a pregnant woman who developed severe proteinuria (5.8 g/day) at 32 weeks of gestation [18]. Measurements of serum sFlt-1 and sEng levels revealed marked elevations, which confirmed that the condition was not due to the exacerbation of lupus nephritis. At 33 weeks of gestation, fetal compromise necessitated emergency cesarean section. The subsequent rapid resolution of proteinuria further supported the diagnosis of hypertensive disorder of pregnancy rather than lupus nephritis. These cases reinforce fluctuations in maternal serum angiogenic factors (an elevated sFlt-1/PlGF ratio, high sFlt-1 and sEng levels, and low PlGF levels) being characteristic of PE and absent in SLE flare, including lupus nephritis. Table 2 summarizes the comparison between these cases and the present case.

**Table 2:** Comparison of previously reported cases with the present case, highlighting the diagnostic utility of the sFlt-1/PlGF ratio in the evaluation of preeclampsia PlGF: placental growth factor, sEng: soluble endoglin, sFlt-1: soluble fms-like tyrosine kinase-1, SLE: systemic lupus erythematosus, w: weeks, d: days.

	Age	Parous	Time of diagnosis		Medication	Termination of pregnancy		Angiogenic factors	Diagnosis
			Hypertension	Proteinuria		Gestational week	Reason		
Case 1 [17]	31	Nullipara	26w	25w	α-Methyldopa (750 mg/day) for hypertension	33w6d	Deterioration of renal function	sFlt-1/PlGF normal: 5.5 (27w), 5.3 (28w) *Confirmed from residual serum after delivery	Diffuse lupus nephritis (renal biopsy)
Case 2 [18]	36	Primipara	None	32w	None	33w4d	Fetal distress and severe proteinuria	sFlt-1 > 95th percentiles: 41.3 ng/mL (33w), sEng > 95th percentiles: 54.8 ng/mL (33w)	Hypertensive disorder of pregnancy
Present case	36	Nullipara	37w	16w	Oral prednisolone (30 mg/day) for proteinuria (>10 g/day) at 31w	37w4d	Fetal distress	sFlt-1/PlGF normal: 1.7 (30w), 4.4 (31w)	SLE flare (31w), preeclampsia (37w)

Table 3 summarizes the typical clinical findings associated with PE, SLE flare, and lupus nephritis. Each condition does not necessarily meet all the criteria listed, and considerable overlap exists. In the present case, apart from severe proteinuria and an elevation in anti-dsDNA antibody levels (later confirmed), there were few characteristic findings of SLE flare. However, the absence of an increased sFlt-1/PlGF ratio provided crucial diagnostic support. Retrospectively, it is also possible that the vesicular rash on the right occipital region, initially diagnosed as herpes zoster, was a manifestation of SLE flare.

**Table 3:** Typical findings of preeclampsia, SLE flare, and lupus nephritis dsDNA: double-stranded deoxyribonucleic acid, PlGF: placental growth factor, sEng: soluble endoglin, sFlt-1: soluble fms-like tyrosine kinase-1, SLE: systemic lupus erythematosus.

	Preeclampsia	SLE flare	Lupus nephritis
Pathogenesis	Systemic vascular endothelial dysfunction secondary to placental insufficiency	Autoimmune disease	
Hypertension	+	±	±
Low platelets	±	+	±
Urinary sediment	－	±	+
Low complements (i.e., C3, C4)	±	+	+
Positive autoimmune antibodies	－	Anti-dsDNA antibody	Anti-dsDNA antibody
Angiogenic factors			
sFlt-1/PlGF ratio	High	Normal	Normal
sFlt-1	High	Normal	Normal
PlGF	Low	Normal	Normal
sEng	High	Normal	Normal

## Conclusions

The assessment of angiogenic factors, particularly the sFlt-1/PlGF ratio, is useful for distinguishing between SLE flare and PE onset in pregnancies complicated by SLE. While SLE flare is often managed pharmacologically, the definitive treatment for PE is delivery, making an accurate differential diagnosis essential. Future research needs to focus on establishing specific sFlt-1/PlGF cutoff values for predicting PE onset, SLE flare, and lupus nephritis in pregnancies complicated by SLE.
